# Influence of Multidimensional Graphene Oxide (GO) Sheets on Anti-Biofouling and Desalination Performance of Thin-Film Composite Membranes: Effects of GO Lateral Sizes and Oxidation Degree

**DOI:** 10.3390/polym12122860

**Published:** 2020-11-30

**Authors:** Bárbara E. Rodríguez, María Magdalena Armendariz-Ontiveros, Rodrigo Quezada, Esther A. Huitrón-Segovia, Humberto Estay, Alejandra García García, Andreina García

**Affiliations:** 1 Advanced Mining Technology Center (AMTC), Universidad de Chile, Av. Tupper 2007, 8370451 Santiago, Chile; barbara.rodriguez@amtc.uchile.cl (B.E.R.); rodrigo.quezada.m@ing.uchile.cl (R.Q.); humberto.estay@amtc.cl (H.E.); 2 Departamento de Ciencias del Agua y Medio Ambiente, Instituto Tecnológico de Sonora, 5 de Febrero 818 Sur, Cd. Obregón, 85000 Sonora, CP, Mexico; maria.armendariz@itson.edu.mx; 3 Group of Synthesis and Modification of nanostructures and Bidimensional Materials, Centro de Investigación en Materiales Avanzados S.C. Parque PIIT, 66628 Apodaca, NL, Mexico; esther.huitron@cimav.edu.mx

**Keywords:** biofouling, TFC membranes, graphene oxide multidimensional sheets, lateral dimensions, oxidation degree, desalination

## Abstract

The influence of the lateral size and the content of graphene oxide (GO) flakes in specific oxygenate functional groups on the anti-biofouling properties and performance of thin-film composite membrane (TFC) was studied. Three different multidimensional GO samples were prepared with small (500–1200 nm), medium (1200–2300 nm), and large (2300–3600 nm) size distribution, and with different degrees of oxidation (GO3 > GO2 > GO1), varying the concentration of the hydrogen peroxide amount during GO synthesis. GO1 sheets’ length have a heterogeneous size distribution containing all size groups, whilst GO2 is contained in a medium-size group, and GO3 is totally contained within a small-size group. Moreover, GO oxygenate groups were controlled. GO2 and GO3 have hydroxyl and epoxy groups at the basal plane of their sheets. Meanwhile, GO1 presented only hydroxyl groups. GO sheets were incorporated into the polyamide (PA) layer of the TFC membrane during the interfacial polymerization reaction. The incorporation of GO1 produced a modified membrane with excellent bactericidal properties and anti-adhesion capacity, as well as superior desalination performance with high water flow (133% as compared with the unmodified membrane). For GO2 and GO3, despite the significant anti-biofouling effect, a detrimental impact on desalination performance was observed. The high content of large sheets in GO2 and small sheet stacking in GO3 produced an unfavorable impact on the water flow. Therefore, the synergistic effect due to the presence of large- and small-sized GO sheets and high content of OH-functional groups (GO1) made it possible to balance the performance of the membrane.

## 1. Introduction

Thin-film composite (TFC) is the most used membrane in desalination plants worldwide [1]. It has a polysulfone (PS) porous interlayer and a polyamide (PA) dense layer [2]. The PS layer is formed by the non-solvent induced phase inversion process [3] and the PA active layer is formed by interfacial polymerization between trimesoyl chloride (TMC) and m-phenylene diamine (MPD) on this PS support [4,5,6].

TFC membranes are very easy to foul due to the physical characteristics of the active layer that make them susceptible to the fouling of organic matter and microorganisms [7,8]. The attachment and proliferation of microorganism communities on the membrane surface can eventually form a biopolymer matrix or complex structure, which is considered to be a biofilm on the membrane surface [9], causing biofouling [10]. The biofouling layer presents an additional barrier to the permeation process, which causes operational problems, leads to the use of higher operating pressures and more frequent chemical cleaning, reduces efficiency, increases the costs of the desalination process, and shortens membrane life [11,12,13,14].

To reduce biofouling problems, many researchers have modified the active layer membrane by adding different antimicrobial materials, such as inorganic nanoparticles (NPs) (Zn, TiO_2_, Ag, Cu, CuO, and Fe, among others) [15,16,17,18,19,20,21,22,23]. Moreover, another antimicrobial material that has gained much acceptance in membrane modification is graphene oxide (GO), as it has antimicrobial properties and increases hydrophilicity [24,25,26,27,28].

GO is a bidimensional material and carbon polymorph with oxygen groups such as carbonyl, carboxyl, or epoxy attached to the basal plane and edges. It is toxic to bacteria because it can generate oxidative stress by producing reactive oxygen species (ROS), causing cellular inactivation, and therefore bacteria death [29,30,31]. Recently, it has been discovered that GO reactivity against bacteria comes from charge transfer and oxidation reactions [32].

Synthesis of GO is usually done by the Hummer method, in which the graphite is oxidized by chemical exfoliation with different acids, and then converted to GO, which obtains an average sheet size of around 5 to 10 µm [33,34]. Modifications in the Hummer method related to changes in precursor exfoliation grade, washing steps, oxidation temperature, acidic medium enhancement, oxidizing agents, and the absence or presence of hydrogen peroxide, among others, have been explored [35]. These proposals have improved the structural properties of the GO, such as a layer with a greater area, a lower number of layers, different oxidation degrees, and sheet sizes [36,37,38,39,40].

In this regard, optimization of the Hummer method remains a challenge in order to obtain GO with properties for specific applications. For instance, the oxidation degree of GO may affect some properties such as antibacterial and surface properties. Thus, high functional group density on the GO surface produces significant cell viability reduction by an increase in ROS production and influence on the hydrophilicity of the material [41,42]. Meanwhile, the GO sheets’ roughness increases with a higher functionalization degree [42,43].

All these features described above have promoted the incorporation of GO into TFC membrane modification and several strategies to incorporate them have been explored, such as self-assembly layer by layer [44,45,46,47,48] and coating by immersion method [49,50,51]. However, low stability of the modification and tendency to agglomerate the GO impacting the uniform dispersion on the membrane surface have been significant concerns when using these strategies [51,52].

Thus, the incorporation of GO during the interfacial polymerization process (IPP) is an alternative method to improve modification stability. Interactions between GO and PA are favored when GO is incorporated during IPP and has a beneficial effect on modification stability. GO can form covalent bonds by condensation reactions with terminal groups of trimesoyl chloride in the linear portion of polyamide, and hydrogen bonding with terminal primary as well as secondary amines of PA [53,54]. In addition, the presence of GO can improve the water transport properties of membranes by two ways. The hydrophilic functional groups (epoxy, carboxyl, and hydroxyl) in GO can assist in the adsorption of water molecules on the membrane surface, and the water molecules can flow through the GO nanosheet channels [54]. These facts demonstrate the relevance of GO surface chemistry through its oxidation degree on modified membrane performance during IPP.

Some research works about the modification of TFC membranes by the incorporation of GO into the PA layer during interfacial polymerization have been reported, showing high water permeability, an anti-biofouling property, and even chlorine resistance. In some cases, a decrease in salt rejection has also been observed. Increase in the water flux have been attributed principally to surface hydrophilic properties conferred by oxygenated functional groups present in GO sheets [28,53]. Thus, enhanced hydrophilicity of the composite GO poly(amide)membranes has been supported mainly by the presence of hydroxyl functional groups on the composite surface, which facilitate water transport through the material [55]. In this regard, controlling OH-group contents in GO to be incorporated into TFC membranes can be an interesting strategy to achieve this benefit. However, few contributions have sought this control.

Moreover, several authors have described that the GO size and GO concentration are key factors to improve the overall performance of the GO-TFC membranes [56,57,58]. Thus, on the one hand, membranes modified with a large size (GO > 10 μm) could show an abnormal surface morphology, detrimentally affecting desalination performance. Whilst GO small fraction (size < 1 μm) could improve water permeability, however, this improvement depends on GO concentration [28,53,54,59]. On the other hand, GO of a size <5 µm could promote antibacterial activity because these large GO sheets cover cells more easily, avoiding their proliferation once fully covered, and resulting in a reduction in cell viability. Meanwhile, small GO sheets promote lower antibacterial activity and tend to form aggregates that could limit ROS production and bacteria death by contact [60]. Hence, the optimization of GO size and concentration should provide a better fit within the active layer, making the water transport through the membrane easier, without affecting the GO antimicrobial properties.

Most of the studies about the modification of TFC membranes by incorporating GO into the PA layer during the interfacial polymerization process have focused on GOs with a lateral dimension of less than 800 nm with areas <1 μm^2^ and optimal concentrations of GO above 40 ppm [28,53,56,57]. Thus, the advantages and disadvantages of the incorporation of GO with large or small sizes have been discussed separately in the literature. Moreover, the GOs employed in these studies have been obtained by post-reaction additional treatment such as breaking up with an ultrasonic probe or by sieving with the membrane, which implies that GO requires an additional treatment after its synthesis to obtain a material with appropriate dimensions.

Furthermore, a significant range of GO lateral size between 800 nm and −5 μm has not been considered to be a focus for study; GO multidimensional sheets within the aforementioned size range also have not been explored. In this last regard, multidimensional sheets of GO with the optimum proportion of large- and small-sized sheets could promote a synergic effect between both sizes and balance their impact on membrane performance.

For this, a balance in the GO synthesis parameters must be found. The aim is to obtain an optimum lateral size control, promote the size multidimensionality of GO, and have a density of oxygenated surface groups controlling the OH-group contents, in order to favor the hydrophilicity and antimicrobial activity, as mentioned above.

Taking these aspects into account, the aim of this work is to explore the modification of the TFC membrane with a low concentration of multidimensional GO sheets (10 ppm), with different lateral size dimensions from 500 to 3200 nm, and different contents of hydroxyl groups incorporated during the interfacial polymerization process. These multidimensional GOs were obtained by applying a method that controlled the lateral size and oxidation degree of sheets in a single step. Thus, the incorporation of multidimensional GO is expected to have a synergistic effect due to the presence of large- and small-sized sheets, in addition to the presence of specific oxygenated surface groups, which will lead to a balance in anti-biofouling and desalination properties.

## 2. Materials and Methods

### 2.1. Materials

All the chemical reagents used for the synthesis of GO were purchased from Sigma-Aldrich, St. Louis, MO, USA. For the synthesis of GO, the mineral graphite was the raw material, while sulphuric acid (H_2_SO_4_, >95%), nitric acid (HNO_3_, 68–70%), and potassium permanganate (KMnO_4_, >97%) were the oxidant reagents, and hydrogen peroxide (H_2_O_2_, 30% *v*/*v*) was used to stop the oxidative process. For the synthesis of TFC membranes, polysulfone (PS, Udel P-3500 MB7 (in pellet form, molecular weight 83,000 g/mol; Solvay Advanced Polymers, Brussels, Belgium)), while 1-methyl-2-pyrrolidinone (NMP, >99.5%) and *N,N*-dimethylformamide (DMF, C_3_H_7_NO, >99%) from Sigma-Aldrich, (Darmstadt, Germany) were used for the PS support formation by the phase inversion method. Trimesoyl chloride (TMC, C_9_H_3_Cl_3_O_3_), m-ohenylenediamine (MPD, C_6_H_8_N_2_), sodium hydroxide (NaOH, >97%), and n-hexane (C_6_H_14_, >95%) from Sigma-Aldrich (Darmstadt, Germany), were used for the interfacial polymerization of PA on PS support.

### 2.2. Synthesis of Graphene oxides (GOs) with Different Lateral Sizes and Oxidation Degrees

The synthesis of the graphene oxide was carried out using the same oxidation methodology for the different lateral sizes tested. Initially, graphite was added to a concentrated solution of H_2_SO_4_/HNO_3_ (volume ratio 3:1) according to the methodology registered by the laboratory for the synthesis and modification of nanostructures and two-dimensional materials [61]. This mixture was sonicated for 30 min at a constant temperature before the oxidation process began. Potassium permanganate was added at a ratio of 1:7 with respect to the mass of graphite. The solution was heated to 70° C and stirred for 24 h. Finally, hydrogen peroxide was added to the solution to stop the oxidation process and it was washed with deionized water until pH = 7. The variation in the amount of hydrogen peroxide used to stop the oxidation reaction allowed us to obtain GOs with different oxidation degree and size sheets distribution, which were identified as GO1 and GO2. The lateral sizes were divided into the following 3 populations: (1) 500–1200 nm, (2) 1200–2300 nm, and (3) 2300–3600 nm. GO1 did not have any treatment after the oxidation process, it was kept in aqueous dispersion and it contained sizes of the 3 populations. GO2 contained lateral sizes of only 2 populations. In this case, the material was dispersed in a methanol/water mixture, in order to separate the flakes due to the effects of interaction with a solvent of polarity other than water, according to [62]. Finally, after obtaining the GO1, the sample was subjected to centrifugation for 1 h at 4500 rpm, in order to have a homogeneous lateral size. Only the sheets contained in the supernatant were recovered to be used later. This material was labeled as GO3 and had a lateral size that falls into a single smallest population.

The most oxidized GO was the one to which the lowest concentration of peroxide was added in the final stage of the process (GO3 > GO2 > GO1).

### 2.3. Synthesis of Thin-Film Composite (TFC) Membranes and GO Incorporation

The TFC membrane consisted of a PA layer on a PS support for the pristine membrane and PA with GO for the modified membrane. The PS layer support was synthesized by the phase inversion method, according to previously reported procedures [15,21]. The PS was dissolved in a solvent mixture of 4:1 DMF/NMP at 65 °C for 2 h to ensure complete PS dissolution. The composition of PS/(DMF/NMP) solution was 15/85 (wt %). The PS was dissolved in a solvent mixture for promoting the regular macro avoid formation in the PS support [63]. DMF was used a principal solvent, and NMP was used as pore former agent. After, the casting solution obtained was uniformly dispersed onto glass plate using a casting knife with the knife gap set at 200 μm (BYK, Geretsried, Germany). The PS support was immersed into water coagulation bath for 1 min at room temperature, and washed with water for 24 h. The PA layer was prepared using the interfacial polymerization method on PS support [23,64]. The PS support was immersed in aqueous MPD (2 wt %) solution for 2 min, and then was immersed in TMC (0.2 wt %) hexane solution for 1 min. After, the membrane was cured at 78 °C for 8 min. Finally, the PA membrane obtained was washed with distilled water and dried at room temperature for 24 h. PA membranes with different GOs (modified membrane) were synthesized by adding the respective GO (10 ppm) to the aqueous MPD solution, previously homogenized by a sonic bath (ISOLAB, GmbHha, Frankfurt, Germany) for 30 min at 25 °C. The modified membranes are referred to as PA+GO1-PS, PA+GO2-PS, and PA+GO3-PS, while the unmodified membrane is referred to as PA-PS.

### 2.4. Characterization of GO with Different Lateral Dimensions

Microscopic characterization was carried out by a field emission scanning electron microscope (FE-SEM) in scanning transmission electron microscopy mode (STEM). The model Nova Nano SEM 200 brand FEI (Hillsboro, OR, USA) was used. To determine the dispersion plot of the GO lateral dimensions, 20 micrographs were analyzed, taking measurements of the sheets’ width and length, from which a population of 61 GO sheets was collected. The histograms were obtained with the Minitab 17 software. Crystalline structure was determined by X-ray diffraction (XRD) using Cu-Kα1 radiation of 1.54059 Å on a PANalytical Empyrean diffractometer (PANalytical Inc., Westborough, MA, USA). Raman measurements were carried out using a LabRAM HR Evolution Raman spectrometer system (HORIBA, San Francisco, CA, USA). The different graphene oxide Raman spectrums were obtained with a wavelength of 633 nm.

The Debye–Scherrer equation was used to calculate the distance between each layer (interplanar distance) formed in the GOs, Equation (1) [65]:

(1) $$\tau =\frac{k\lambda }{\beta \cos\theta }$$

where τ is the mean size of the ordered (crystalline) domains and *k* is a form factor without dimension, with a value close to the unit. The form factor has a typical value of about 0.9; *λ* is the wavelength of X-rays; *β* is the enlargement of the line to half the maximum intensity of the wavelength, in radians; and *θ* is the Bragg angle (the angle between the incident X-ray beam and the crystal diffraction planes).

X-ray spectroscopy (XPS) analysis was performed with a Thermo Fisher Scientific Escalab 250Xi spectrometer (Portland, OR, USA) using a monochromatic Al Kα source (1486.68 eV) and an analysis area of 1.33 mm^2^. The sample spectrum was obtained by measuring, at high resolution, the regions corresponding to C1s, N1s, and O1s binding energies with 20 eV, a take-off angle of 45°, and a step analysis of 0.1 eV. Functional groups were confirmed by FTIR spectra recorded on an FT/IR-4100 Jasco spectrophotometer (Tokyo, Japan) using KBr discs. The zeta potential of GO materials was measured by a Zetasizer Nano-ZS de Malvern Instruments (Birmingham, United Kingdom) at room temperature in 1 mM NaCl as background condition. The pH was controlled by titrating 0.25 mol L^−1^ NaOH and 0.25 mol L^−1^ HCl into the electrolyte solution.

### 2.5. Characterization of GO-Modified Membranes

GO presence in membranes was confirmed by Raman spectra. Raman measurements were obtained using a LabRAM HR Evolution Raman spectrometer system (HORIBA, San Francisco, CA, USA). The spectra were obtained with a wavelength of 633 nm. The surface morphology of the membranes was analyzed by atomic force microscopy (AFM) (Asylum MFP 3D-SA AFM microscope, Oxford Instruments, Abingdon, England) using 1 cm^2^ of the membrane sample. The hydrophilicity of membranes was determined using the contact angle system, OCA, 15 plus (DataPhysics Instruments, Filderstadt, Germany). The membrane surface composition was characterized by X-ray photoelectron spectroscopy (XPS) Thermo Fisher Scientific Escalab 250Xi spectrometer (Portland, OR, USA), operated with a conventional Al Kα source. Each spectral region was scanned for three different zones. The concentration of elements C1s, N1s and O1s were determined in order to calculate the crosslinking degree (X) for each membrane using the following formulae [66,67]:

(2) n+m=1

(3) $$\frac{N}{O}=\frac{3m+2n}{3m+4n}$$

where *N* and *O* are the nitrogen and oxygen element concentrations measured by XPS, respectively, m is the ratio of the crosslinking part and n is the ratio of the linear part in the polyamide.

### 2.6. Anti-Biofouling Test

The biocide effect of the membrane was tested by the immersion method in an enriched solution with *Escherichia coli* (*E. coli*). This enriched solution was elaborated following Garcia et al.’s methodology [21]. *E. coli* solution was prepared using tryptone soya broth (TSB, 30 g L^−1^) as the culture medium; it was diluted in phosphate-buffered saline (PBS) to 1 × 10^7^ cell mL^−1^. A piece of the membrane (approximately 4 cm^2^), previously sterilized, was immersed into the enriched solution and incubated at 30 °C for 4 h. To determine the surviving *E. coli* population in the enriched solution after membrane immersion, the serial dilution (1:10) method was used; dilutions were prepared up to 10^−5^ using PBS as the diluent. Then, 1 mL of the highest dilution was spread on Petri dishes containing LB agar and incubated for 24 h at 37 °C by triplicate. 

The bacteria adhesion onto membranes was assessed by live and dead methodology. After being incubated for 4 h, the membranes were removed from the enriched solution and washed with aqueous NaCl solution (0.85 wt %). Membranes were cut (1 cm^2^) to evaluate bacterial viability using a bacterial viability kit (Molecular Probes, LIVE/DEAD BacLight L7007). Results were analyzed by an epifluorescence microscope (Zeiss, AxioLab A1, Jena, Germany) with a 100× objective.

### 2.7. Membrane Performance Test

To determine membrane performance, the permeate flux and salt rejection was calculated according to Garcia et al.’s methodology [21,68]. Before the tests, the membranes were compacted for 1 h at 300 psi using distilled water. The permeate flux and salt rejection was assessed in a CF042 crossflow cell unit (Sterlitech Corporation, Kent, WA, USA) with an effective membrane area of 0.0042 m^2^ in a feed solution of 1000 mg L^−1^ NaCl at 300 psi according to Saleh and Gupta [69] using a diaphragm pump of positive displacement (Hydra-Cell, M03SASGSNSCA, Wanner Engineering, Minneapolis, MN, USA). Pressure was obtained using pressure transmitters (PX9111). The test was run for 130 min at 25 ± 1 °C, and each experiment was performance in triplicate. The permeate flux was calculated by Equation (2) [21,68]: J = V/A ∆t (1), where J (L m^−2^ h^−1^) is the membrane flux, V (L) is the volume of permeated water, A (m^2^) is the membrane area and ∆t (h) is the permeation time.

Additionally, solute rejection was measured from the feed and permeate solution. The permeate flux and salt rejections were calculated by Equation (2) [70]: Rejection = (C_f_ − C_p_)/C_f_ × 100 (2), where C_f_ and C_p_ are the concentrations of the feed solution and permeate solution, respectively. The concentrations of these two solutions were obtained by their respective conductivity measurements.

## 3. Results and Discussion

### 3.1. GO Characterization

**Figure 1:**
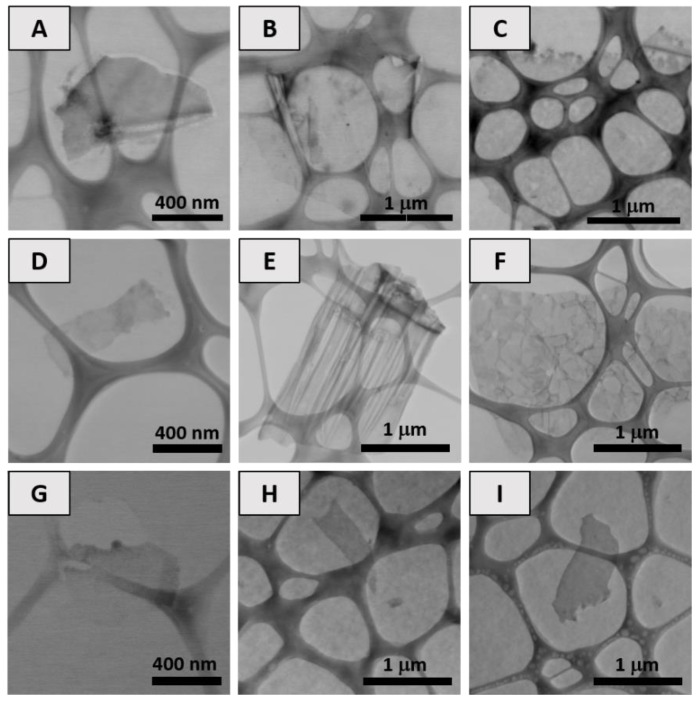
SEM-STEM micrographs for the different synthesized graphene oxides. (**A**–**C**) GO1, (**D**–**F**) GO2, and (**G**–**I**) GO3.

Figure 1 shows STEM images of different GOs synthesized. For all cases, it is possible to observe a sheet structure with high transparency, wrinkles, and a rolled edge. The sizes of different synthesized GO sheets are higher than 450 nm but lower than 5 µm in two sheet axes (Figure 1).

**Figure 2:**
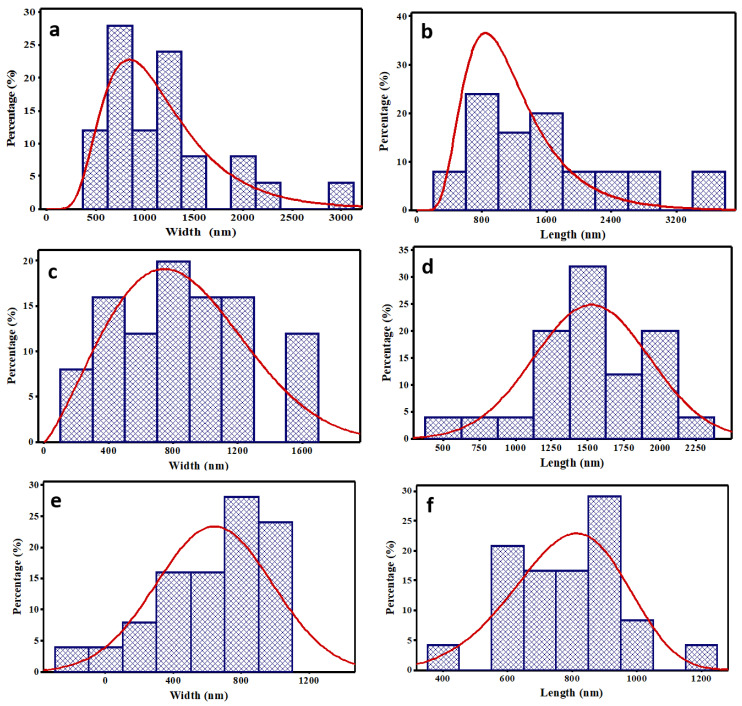
Dispersion plot of GOs’ lateral dimensions. (**a**) GO1 width; (**b**) GO1 length; (**c**) GO2 width; (**d**) GO2 length; (**e**) GO3 width; (**f**) GO3 length.

A more detailed study of GO sheet sizes obtained dispersion plots of the size distribution of sheets for two dimensions (width and length), which are shown in Figure 2. It is possible to observe three principal groups of sizes, i.e., Group 1 with small-sized sheets between 500 and 1200 nm, Group 2 with medium-sized sheets between 1200 and 2300 nm, and Group 3 with large-sized sheets between 2300 and 3600 nm.

The percentages of sheet populations with different sizes for each GO are shown in Table 1. The GO1 sheets have a broad and heterogeneous size distribution, with lateral dimensions within the three size groups (Figure 2a,b) and an average area of sheets of around 2.14 µm^2^. Seventy-four percent of GO1 sheets have a width with sizes within group 1, while the remaining 18% and 8% are contained within Groups 2 and 3, respectively (Table 1). Similarly, a high percentage (48%) of GO1 sheets present a length with sizes within group 1, with 28% and 24% contained within Groups 2 and 3, respectively (Table 1). Thus, the GO1 size distribution confirms its multidimensional behavior with heterogeneous distribution between the three size groups.

Likewise, the GO2 sheets show a heterogeneous but narrower size distribution, with lateral dimensions within only two size groups (Figure 2c,d) and an average area of around 1.94 µm^2^. In this case, 88% of GO2 sheets have a width with sizes within Group 1, while the remaining 12% is contained within Group 2 (Table 1). Regarding length, 88% of GO2 sheets have sizes within Group 2, while only 12% are within Group 1 (Table 1). Thereby, GO1 and GO2 are multidimensional sheet material formed by small-width sheets with a high percentage within Group 1, although a notable difference in the length of GO2 sheets is observed principally formed by medium-sized sheets within Group 2.

GO3 sheets have smaller sheet sizes between 500 and 1200 nm, with 100% of sheets contained in Group 1 for both lateral dimensions (Figure 2e,f). Its sheets present an average area of around 1.28 µm^2^. Remember that GO3 was separated by centrifugation, a post-treatment to the oxidation process before use.

Thus, the main difference between GO sizes lies in the length of their sheets. The length of GO1 sheets has a size distribution that is broad and heterogeneous, with sheets in all size groups, whilst GO2 length is almost entirely contained in the medium-sized group (Group 2) and GO3 length is totally contained within the small-sized group (group 1).

**Figure 3:**
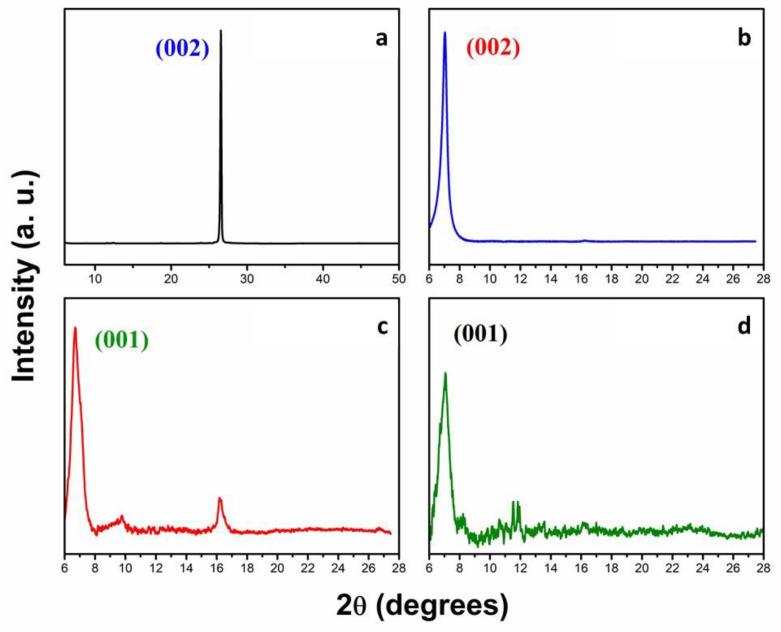
X-ray diffraction (XRD) pattern. (**a**) Graphite, (**b**) GO1, (**c**) GO2, (**d**) GO3.

XRD diffractograms for synthesized GOs and graphite are given in Figure 3. Graphite has one characteristic plane and corresponds to the basal reflection plane (002) at 26.51° (2θ) with an interlayer spacing of 0.34 nm [71]. After the oxidation process, the (002) crystallographic plane in the different GOs shows an evident shift to lower angles (2θ). The shift is attributed to an increase in the interlayer spacing, assigned to the C-axis expansion from the introduction of oxygen functional groups (epoxy, carboxyl, and hydroxyl groups) between layers and it corresponds to (001) plane [72]. The spacing was calculated from Equation (1) obtaining interlayer distances of 0.915 nm, 0.999 nm, and 1.162 nm for GO1, GO2, and GO3, respectively. Other peaks, similar to those present in GO2 at 9.8 and 16.7 (2θ), are due to distorted lattice. These peaks correlated with those observed in [62], which mentioned that the presence of methanol-like solvent in GO dispersions produced structural changes in it.

The identification of functional groups and the C/O atomic ratio estimation was achieved by XPS analysis of the different GO samples (Table 2). GO1 has a C/O atomic ratio of around 2.09. The binding energies corresponding to the C1s high-resolution analysis are attributed to sp^2^ and sp^3^ hybridized carbon (284.5 eV), hydroxyl groups (285.4 eV), carbonyl groups (287.2 eV), and carboxyl groups (288.7). Meanwhile, for the O1s spectrum, the obtained functional groups were hydroxyl and carboxyl (C–OH and –COOH). The percentages for each group present in C1s were 3.2% (C–C, C=C), 45.7% (C–O), 38.6% (C=O), and 12.5% (O–C=O), respectively. According to the Lerf–Klinowski model, the GO presented with a predominance of epoxy and hydroxyl groups in the basal plane, and carbonyl and carboxyl groups at the edges [73]. For the case of GO1, there was a predominance of hydroxyl groups in the basal plane.

The GO2 sample shows a C/O atomic ratio of around 1.96. Similar to the previous case, the C1s binding energies correspond to sp2 and sp3 hybridized carbon (284.5 eV), hydroxyl groups (285.4 eV), epoxide groups (286.8 eV), and carboxyl groups (288.7). The percentages for each present group were 13.2% (C–C, C=C), 26.1% (C–O), 45.9% (C–O–C), and 14.8% (O–C=O), respectively. The GO2 sheets have a predominance of hydroxyl and epoxide groups in the basal plane, and a lower quantity of carbonyl groups at the edges with respect to GO1.

The C/O atomic ratio of GO3 is found to be around 1.77, which is a typical atomic ratio value for a method with a high oxidative level [33]. The GO3 sheets presented a predominance of hydroxyl and epoxide groups in the basal plane and a lower quantity of carbonyl groups at the edges. The percentages for each present group were 14.87% (C–C, C=C), 22.95% (C–O), 50.5% (C–O–C), and 11.63% (O–C=O), respectively. The binding energies for C1s and O1s of GO3 are reported in Table 2.

According to the C/O ratio, an increase in the oxidation degree of GOs is related to the decrease in this value. Thus, material that presents the highest oxygenated group density has the lowest C/O ratio following the same trend as GO3 > GO2 > GO1. GO2 and GO3 both have hydroxyl and epoxy groups on their surfaces but GO3 presents the highest content of epoxy groups. Meanwhile, GO1 presents only hydroxyl groups on its surface. 

It is known that the oxygenated functional groups present at the GO surface impact surface properties such as roughness and their antibacterial activity. The roughness of the GO sheets increases when lateral size diminishes and oxidation degree increases [42,74]. Additionally, oxidative stress is a key mechanism for the antibacterial activity of GO through ROS generation [29], while ROS production from GO is achieved by the presence of the oxygen functional group. In this regard, previous studies have shown that the functional group density at the GO surface directly affects cell viability. High functional group density on the surface of the GO produces significant losses of cell viability [41]. 

**Figure 4:**
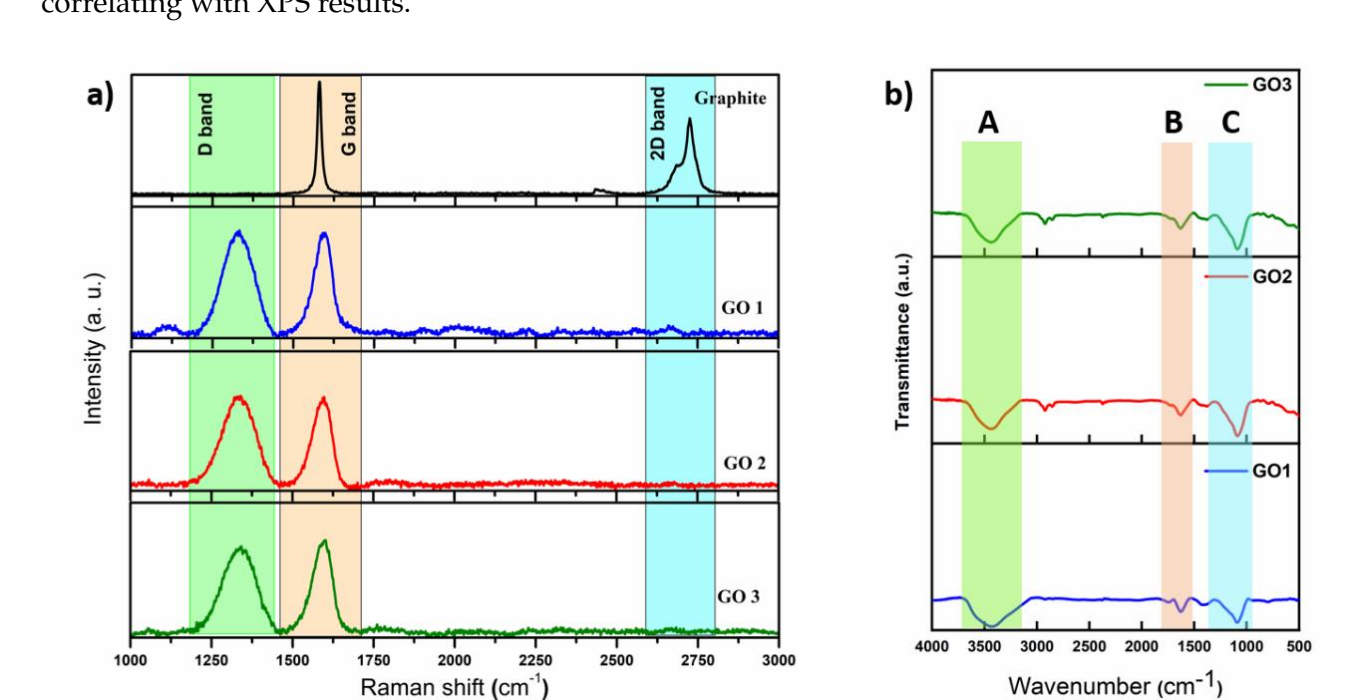
(**a**) Raman spectra of GOs synthesized; (**b**) FTIR spectra of GOs synthesized.

Raman spectra for synthesized GOs are shown in Figure 4a. The D band values for the different GOs are located at 1329, 1334, and 1336 cm^−1^, and the G band at 1591.5, 1590.8, and 1591.3 cm^−1^ for GO1, GO2, and GO3, respectively. The D band was observed due to an induced disorder in the graphite after the oxidation process, caused by the presence of oxygen functional groups, vacancies, grain boundaries, symmetry breaking, and other defects. According to different reports, the Full Width at Half Maximum (FWHM) value of this band increases with a higher oxidation level and defects present. The values of FWHM were 112, 117, and 119 for GO1, GO2, and GO3 respectively, which denotes a correlation with the obtained XPS results. The G band of the different GO shifted between 10 cm^−1^ and 11.5 cm^−1^ with respect to the G band of graphite, which was attributed to the oxidative process. The graphite spectrum presents a peak associated with the G band (1580.1 cm^−1^) due to the stretching of the C-C bond of sp^2^ carbons, and therefore shifts at this band are linked to a decrease in sp^2^ carbons hybridization and the presence of sp^3^ carbons once the graphite is oxidized. Another important feature present in graphite is the two-dimensional (2D) band and it is used to evaluate the structural parameters of the c-axis orientation. In Figure 4a, one can observe the disappearance of the 2D band after oxidation, which indicates a symmetry breaking in the c-axis and a high oxidation level of the raw material. Finally, the variation in the I_D_/I_G_ ratio with respect to the oxidation level did not present considerable differences between the obtained GO. According to the literature, lower oxidation levels result in an increase in the I_D_/I_G_ ratio, which decreases with increasing oxidation and finally becomes saturated at higher oxidation levels [75]. In our case, the results by XPS indicated high oxidation levels between 1.77 and 2.09, and the values for I_D_/I_G_ ratios were 1.00, 1.01, and 1.0 for GO1, GO2, and GO3, respectively, which represents saturation in the oxidation level.

Functional groups in GOs were identified by FTIR spectra analysis; these spectra are shown in Figure 4b. Three band groups can be observed. Group A (3200–3400 cm^−1^) is a highly broadened band and corresponds to the presence of hydroxyl groups and absorbed water. In this zone, the band observed for GO1 showed the highest intensity which was attributed to a high amount of -OH groups present in GO1. Group B contains two bands as follows: one band at 1720 cm^−1^, which is attributed to stretching vibrations of the (–COOH) group corresponding to the carboxyl and carbonyl groups, and the second band at 1627 cm^−1^, assigned to in plane vibration (C=C) from unoxidized sp^2^ bonds. Group C contains bands within 980–1250 cm^−1^, corresponding to C–O stretching vibration modes correlating with XPS results.

### 3.2. Characterization of GO-Modified Membrane

**Figure 5:**
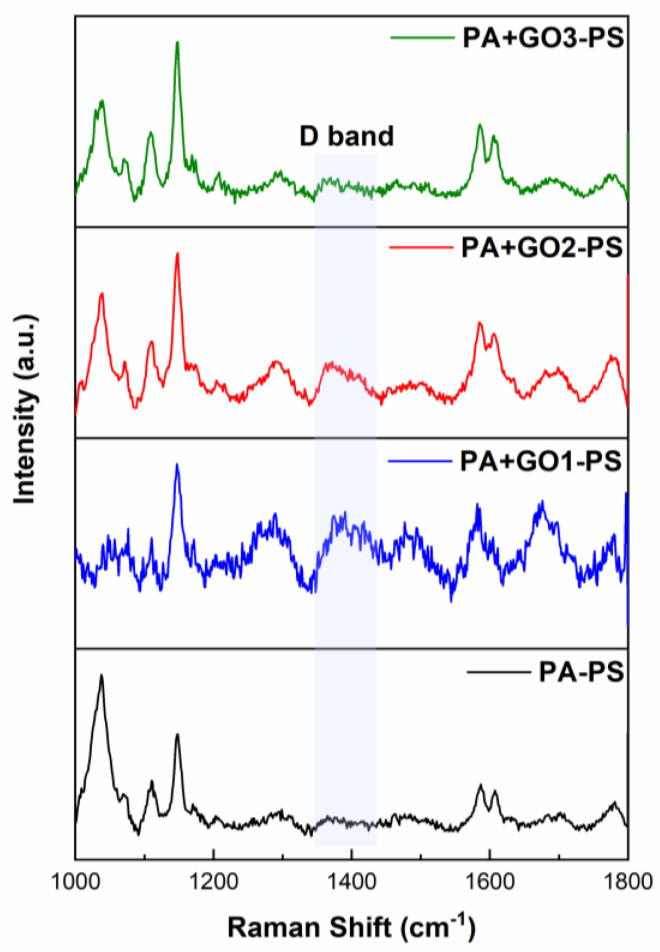
Raman spectra of membranes.

Synthesized GOs were incorporated into the active polyamide layer during the interfacial polymerization process to produce PA+GO1-PS, PA+GO2-PS, and PA+GO3-PS modified membranes. The presence of GO in the PA layer of modified membranes was confirmed by Raman spectra (Figure 5). The Raman spectrum of the PA-PS membrane without GO shows five principal bands. Two bands at 1078 cm^−1^ and 1112 cm^−1^ correspond to symmetric and antisymmetric SO_2_ stretching, respectively [57]. Previous studies have indicated that the vibration of SO_2_ groups can be observed because the PS is the thickest layer in the membrane [57]. The other three bands observed at 1149 cm^−1^ and 1587–1620 cm^−1^ are characteristic of the TFC membrane and originate from symmetric C–O–C stretching and phenyl ring vibration, respectively [24]. The Raman spectra of modified membranes show two broad bands around 1360 and 1600 cm^−1^, which are attributed to the D and G peaks of GO, respectively (Figure 5). The D peak was observed in all Raman spectra of modified membranes (see the blue zone in Figure 5); however, this peak presented a lower intensity in the PA+GO3-PS membrane, which could suggest that the PA+GO3-PS membrane surface has an inhomogeneous distribution of GO3 sheets. The aforementioned feature could generate a detrimental impact on the desalination performance of the PA+GO3-PS membrane.

**Figure 6:**
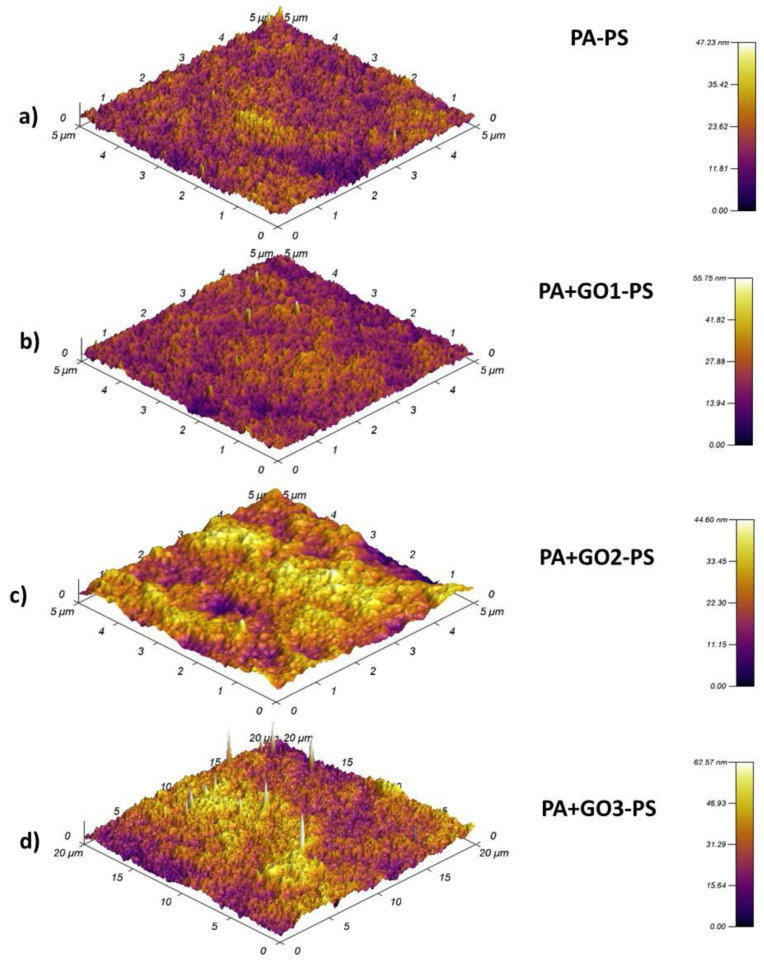
Atomic force microscopy (AFM) images of membranes. (**a**) PA-PS, (**b**) PA+GO1-PS, (**c**) PA+GO2-PS, (**d**) PA+GO3-PS.

The surface characteristics of membranes, such as roughness and hydrophilicity, were studied by AFM and contact angle, respectively. Figure 6 shows the AFM images of the pristine membrane and modified membranes. The pristine membrane exhibits characteristics consistent with those of interfacially polymerized polyamide membranes, which consist of ridge-and-valley layers (Figure 6a) [71,76]. However, the incorporation of different GOs produced changes in the ridge-and-valley layer distribution of the top surfaces, and these changes affected the surface roughness of modified membranes. Thus, the root mean square roughness parameter was increased for modified membranes as compared with an unmodified membrane PA+GO3-PS > PA+GO1-PS > PA+GO2-PS > PA-PS (Table 3). This result could create bacteria niches, increasing the probability of adhesion onto membranes [77].

It is important to observe that the roughness of the GO sheets increases with their size and oxidation degree and that these features affect the roughness of materials where GO is incorporated, as was observed in modified membranes by [42].

The incorporation of GO1 and GO3 during the interfacial polymerization process produced membranes that were 2.9 and 3.3 times rougher than the pristine membrane, respectively. The AFM images of PA+GO1-PS and PA+GO3-PS (Figure 6b,d) show surfaces with ridge-and-valley layers disrupted by zones with higher peaks similar to nano knives.

**Scheme 1:**
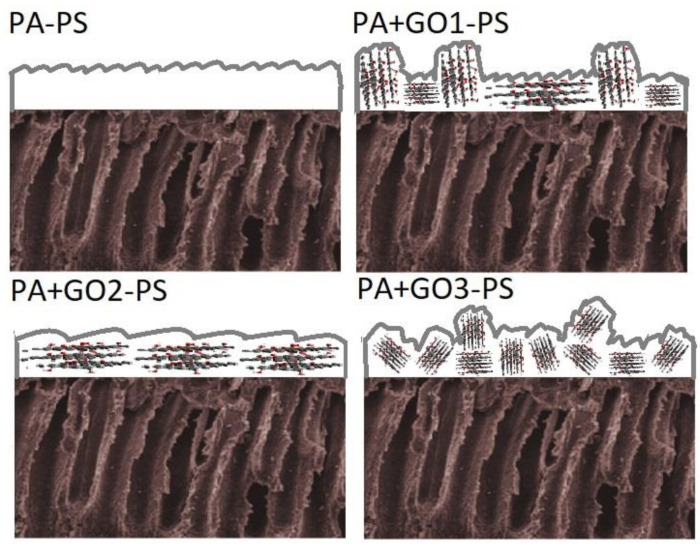
Graphical representation of GO sheets in the polyamide layer of the modified membrane.

Thus, the increase in roughness of the PA+GO1-PS membrane surface might be attributed to the presence of certain large-size sheet populations producing these nano knife zones on the surface, affecting the roughness parameter, as it is shown in Scheme 1. The AFM image of PA+GO1-PS (Figure 6b, Scheme 1) shows that the surface of this membrane has regular and homogenous ridge-and-valley layer distribution except for these nano knife zones. This fact could suggest that the polydispersity of GO1 sheet size promoted good dispersion and exfoliation of the sheets, which limited the formation of the aggregates.

The surface of the PA+GO3-PS membrane shows the highest roughness, although the incorporated GO3 contains smaller-size sheets. In fact, the PA+GO3-PS membrane surface shows more zones with higher peaks similar to nano knives with respect to the PA+GO1-PS membrane surface (Figure 6b,d). The greater roughness observed in the PA+GO3-PS membrane has been attributed to the high oxidation degree of GO3 and to a low redispersion of the sheets, as this GO was lyophilized and redispersed before being incorporated into the membrane. This condition promotes the re-stacking of the sheets, decreasing their effective exfoliation and increasing the membrane roughness through the formation of agglomerates (see Scheme 1).

In contrast, the GO2 incorporation during the interfacial polymerization process affected (in minor grade) the roughness of the modified membrane (1.5 times rougher than the pristine membrane), despite the fact that GO2 has the highest number of sheets with medium-size length (Figure 6c and Scheme 1). However, this result shows that GO2 sheets have a different effect on the height of PA ridges formed during the interfacial polymerization process with respect to GO1 and GO3 materials, which could correspond to a detrimental formation of the polyamide layer as a consequence of GO2 incorporation.

Firstly, the retardation of MPD diffusion into the organic solvent by the GO2 nanosheets could be promoted. During interfacial polymerization, MPD diffusion usually leads to ridge formation. In this case, the GO2 with a high population of significant length would block the MPD diffusion more efficiently and promote a reduction in the number and height of ridges formed (Figure 6c). This behavior has previously been observed by the incorporation of non-fractionated larger-size GO [57,58]. In addition, it is important to note that only GO2 was dispersed in a methanol/water mixture. The presence of catalytic amount of methanol during interfacial polymerization could conduce to structural changes of monomers. For example, the methanol could react with acyl chloride groups of TMC producing the attaching additional methyl groups onto the aromatic rings of the monomer, which could impact on structural parameters of the polymer chain, such as the crosslinking degree and the height of PA ridges [78]. Thus, the formation of polyamide and its crosslinking degree could be affected by the presence of GO2, given by the blockage in the diffusion of the MPD attributed to its size and the reactivity of the TMC with the MeOH used in the dispersion of this GO.

Considering the aforementioned statements, XPS analysis were conducted in order to confirm them. The chemical composition on membranes surface was estimated by XPS and crosslinking degrees were calculated. The results are shown in the Table 4.

**Scheme 2:**
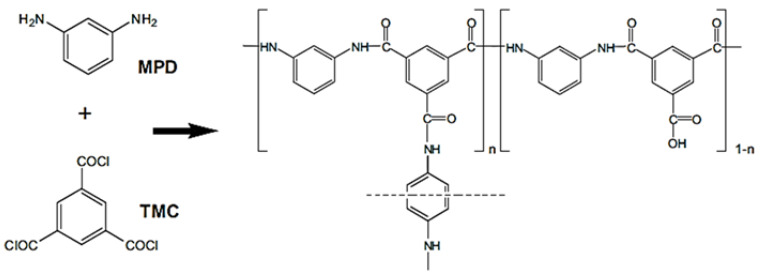
Interfacial polymerization of crosslinked polyamide dense layer based on trimesoyl chloride (TMC) and m-phenylene-diamine (MPD). When *n* = 1, the resulting polymer is fully crosslinked. Every acid chloride monomer is linked with 1.5 amine monomers to give a molecular formula of C_6_H_4_ON. When *n* = 0, the resulting polymer is linear. Every acid chloride monomer reacts with one-amine monomers to give a molecular formula of C_15_H_10_O_4_N_2_.

The PA layer is formed by an interfacial polymerization process to form a crosslinked thin layer of several hundred nanometers thick [79]. In this research, the aromatic PA composite membranes are formed between TMC and MPD monomers (Scheme 2) which have been the most commonly used monomers [79]. The theoretical O/N ratio is 1.0 when the PA layer is fully crosslinked (*n* = 1), because all the O and N atoms are associated with the amide groups to give a 1:1 ratio. This ratio becomes 2:1 for linear polyamide with no crosslinking (*n* = 0). Thus, the synthesized membranes showed an O/N ratio close to 1 indicating that they have high crosslinking degree. Specific crosslinking degrees were calculated using Equations (2) and (3) (Table 4).

The unmodified membrane (PA-PS) showed a crosslinking degree of 78.5% and this value was according to a previous report for TFC membrane [80]. The PA+GO2-PS membrane showed a 8.5% decrease in the crosslinking degree with respect to membrane pristine, confirming the impact on the formation of polyamide when GO2 is incorporated. In contrast, the incorporation of GO1 and GO3 produced membranes more crosslinked than the pristine membrane, and PA+GO3-PS had the highest crosslinking degree. In addition, the thickness of the polyamide layer of unmodified and modified membranes was determined by cross-section SEM images (see ESI). The same trend was observed. Thus, PA+GO2-PS membrane showed the lowest polyamide thickness in correlation with the XPS results.

Overall, the increase in roughness observed in modified membrane surfaces could initiate a higher microorganism adhesion onto the membrane surface, as higher roughness creates favorable conditions for bacteria to settle down more easily, thereby causing their growth and reproduction on the membrane surface [81]. Moreover, the changes observed to the crosslinking degree of modified membrane could impact on their performance.

In addition, the hydrophilicity of modified membrane surfaces was estimated by contact angle measurements (Table 3). Thus, the hydrophilicity of the PA+GO1-PS membrane showed a moderate increase with respect to the pristine membrane. This improvement might be attributed to specific functional groups on the GO1 surface. GO1 showed the highest C/O ratio; hence, it had lower oxygenated functional group density. This GO contains only hydroxyl groups on its surface, just as XPS observed, which can assist in the adsorption of water molecules on the membrane surface. This behavior is according to the results observed in the modified membrane surface with GO by interfacial polymerization [53,82].

Moreover, the PA+GO3-PS membrane showed a more hydrophilic surface with a contact angle of 23.7° (65% lower than the pristine membrane). This can be mainly attributed to the material GO3, which presents the highest oxygen functional group density because it has the lowest C/O ratio and smaller size sheet, and these features favor water adsorption [58]. Moreover, GO3 promotes a located stacking that produces the formation of zones with high peaks similar to nano knives (Scheme 1). The presence of these nano knives could favor the exposition of oxygenate groups increasing the hydrophilicity of PA+GO3-PS membrane.

On the other hand, the contact angle of the PA+GO2-PS membrane was within a broad interval (57°–77°), which indicated that the membrane might have hydrophilic and hydrophobic zones. GO2 sheets showed the longest length. It is known that large GO sheets have more sp^2^ hydrophobic carbon [60], which might promote the differences observed in the hydrophilic character of the modified membrane surface.

### 3.3. Anti-Biofouling Effect of the GO-Modified Membrane

**Figure 7:**
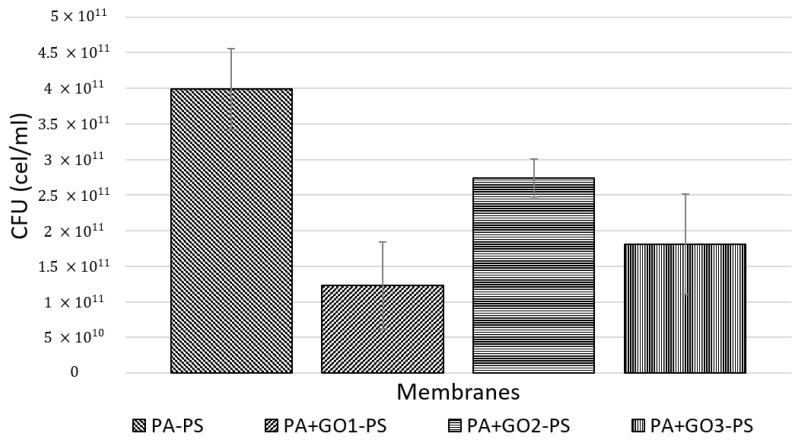
Bactericidal effect of the membranes on *Escherichia coli*.

Bactericidal and anti-adhesion tests were carried out on modified and unmodified membranes (see Supplementary Materials), to evaluate the influence of GO incorporation within the membrane on anti-biofouling properties. The graph shown in Figure 7 denotes the bactericidal effect of the modified membranes in contact with *E. coli*. From this figure, it is possible to observe a significant reduction in the number of *E. coli* colonies in the presence of modified membranes. The results also show that the intensity of the bactericidal effect was dependent on the built-in GO (Table 5). Thus, the bactericidal effect observed in modified membranes can be attributed to the influence of specific oxygenate functional groups from GO sheets. It is known that the oxygenate functional groups promote the production of reactive oxygen species (ROS) [83] and these generate oxidative stress causing cellular inactivation, and therefore bacteria death [29,30,31].

Thus, the PA+GO1-PS membrane showed the highest bactericidal effect with a 69% decrease in colony-forming units (CFU) with respect to the pristine membrane (Table 5). The GO1 has the highest C/O ratio as compared with GO2 and GO3 (Table 2), and thus the lowest oxygenated group density, but GO1 presents only hydroxyl groups on its surface. It is known that hydroxyl groups on the material surface favor ROS generation [84]. Hence, the efficient bactericidal effect observed by the PA+GO1-PS membrane could be attributed to the presence of a high amount of this functional group (45.7%) available on the GO1 surface. Moreover, according to AFM analysis, the uniform dispersion and exfoliation of the sheets and low aggregate formation achieved by the heterogeneity of GO1 sheet sizes also favored the OH groups exposition on material surface.

Likewise, the PA+GO3-PS membrane showed a good bactericidal effect with a 55% decrease in CFUs with respect to the pristine membrane (Table 5). The GO3 sheets have the highest oxygenated group density, but lower available OH groups than observed on the GO1 surface. Moreover, the staking of small-size GO3 sheets led to the formation of agglomerates. Previous studies have indicated that GO sheet aggregation detrimentally affected the bactericidal effect of this material due to the blocking of available oxygenated groups [30]. In contrast, the PA+GO2-PS membrane showed the lowest bactericidal effect, which might be attributed to low oxygenated group density generated by the presence of sheets with a longer length in GO2. Considering these trends, an interesting future work would be the determination of ROS from membranes modified by the incorporation of GOs and its correlation with the functional groups observed in them.

**Figure 8:**
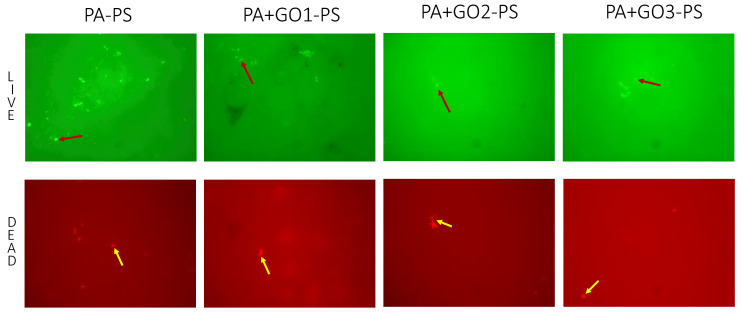
Fluorescent bacteria images of membranes with the dead/live detection kit.

**Figure 9:**
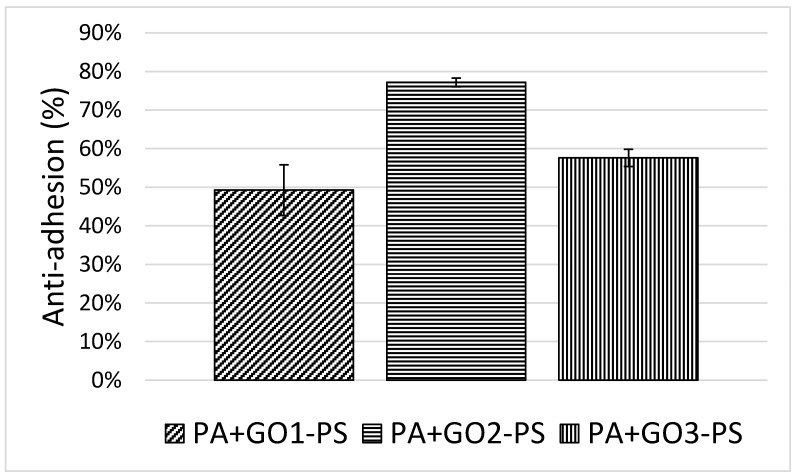
Anti-adhesion performance of membranes against *E. coli*.

The anti-adhesion capacity of modified membranes was estimated by the number of bacteria cells adhered to the membrane surfaces. These results are shown in Figure 8 and Figure 9 and Table 5. It is possible to observe that the anti-adhesion capacity was also dependent on the GO incorporated and this increased as follows: PA+GO1-PS < PA+GO3-PS < PA+GO2-PS. 

It has been reported that anti-adhesion capacity of polymeric membranes can be influenced by the surface charge of the membrane surface. A negative surface charge favors the anti-adhesion property of the membranes because it favors the electrostatic repulsion between the membrane and the bacteria, as *E. Coli* [21]. Thus, zeta potential of GO materials can affect the surface properties of GO-polymeric modified membranes [85,86]. In this regard, experiments were conducted for determining the zeta potential of different GOs used in this study. The results are shown in the Table 6.

The zeta potential for GO1, GO2, and GO3 was determined within a specific pH interval (7–9). This pH interval includes the pH of seawater [18] and the anti-biofouling experiments conditions. The GO1 showed a zeta potential close to a neutral value. Thus, the PA+GO1-PS membrane surfaces showed a moderate anti-adhesion effect with a 49% reduction in total adhering cells (Figure 7, Table 5). Hence, the GO1 should not contribute to improve the anti-adhesion of the PA+GO1-PS layer by electrostatic repulsion. In addition, the significant increase in roughness of the PA+GO1-PS membrane could promote more favorable conditions for bacteria adhesion.

In contrast, GO2 and GO3 showed similar and negative zeta potential, which would favor the phenomenon of electrostatic repulsion. Thus, the PA+GO2-PS and PA+GO3-PS membrane surfaces showed a high and moderate reduction of 77% and 58% in total adhering cells, respectively. The anti-adhesion effect showed by the modification with GO2 could be attributed to the combined effects of the negative zeta potential of GO2 and the lower surface roughness for PA+GO2-PS. Whilst GO3, in spite of the negative zeta potential, the anti-adhesion capacity of PA+GO3-PS membrane is affected by the significant increase in its roughness, considering that biofouling is carried out by entrapment in membranes with rougher topologies [87].

### 3.4. Desalination Performance of the GO-Modified Membrane

Membrane performance tests were carried out using a crossflow system. Table 7 shows the permeate flux and rejection percentage of the unmodified membrane and modified membranes. It is possible to observe that the performance of the membranes depends on the incorporated GO. Thus, several factors, such as hydrophilicity, sheet size, the types of oxygen groups present in sheets, crosslinking degree, and the absence or presence of stacking, impact membrane performance.

Thereby, the PA+GO1-PS membrane showed the best desalination performance with a permeate flux of 26 ± 3 L m^−2^ h^−1^ indicating a 136% increase with respect to the unmodified membrane. Hence, modification with GO1 in the membrane promotes an increase in the permeate flux on the membrane. This result agreed with those reported by some researchers, who found that GO sheets increased the flux in TFC membranes [83,87,88]. The multidimensional graphene oxide GO1 contains a greater amount of hydroxyl group on its surface, which favors water adsorption. Moreover, the heterogeneous size distribution of GO1 sheets with a low proportion of larger sheets efficiently exfoliated sheets and limited the staking of sheets, producing a better assembly between GO1 and the PA layer, and promoting the formation of channels for water preferential flow.

In contrast, the GO2 and GO3 incorporation detrimentally affected the modified membrane performance. The PA+GO2-PS membrane showed the presence of hydrophobic zones on the modified membrane surface, as was discussed for the contact angle, which impacted the water flux, producing a significant reduction in the permeate fluxes of the PA-GO2-PS membrane (81.8% lower than the pristine membrane) (Table 7), considering that the hydrophobicity affects transport properties through the membrane [89]. The PA+GO3-PS membrane showed a drop in water flux (54.5%) and a significant reduction in salt rejection. The GO3 presents a homogeneous and smaller size. However, this material was submitted to post-treatment before being incorporated into the membrane, and this process promoted re-stacking of the sheets. Consequently, the stacked GO3 sheets detrimentally affected the membrane surface properties, preventing water from flowing through it [57]. In addition, the PA+GO3-PS membrane presented the highest crosslinking degree of surface (Table 4) forming a denser layer that could decrease the water pass.

## 4. Conclusions

The incorporation of a low concentration (10 ppm) of graphene oxide multidimensional sheets into the polyamide layer of the TFC-RO membrane during the interfacial polymerization process influenced their anti-biofouling properties and desalination performance, which was associated with the lateral size and oxidation degree of the built-in GO. The GO sheet sizes, the types of oxygen groups in the GO sheets, and the absence or presence of stacking are the parameters that directly influence membrane desalination and anti-biofouling performance.

Thus, a balance of the membrane performance on anti-biofouling and desalination properties was achieved by GO1 incorporation. The PA+GO1-PS membrane showed an excellent bactericidal effect, good anti-adhesion capacity, and a significant increase in water flux with respect to the unmodified membrane. Specific features of GO1, such as heterogeneous-size sheet distribution with sizes between 500 and 3200 nm, low sheet staking, and a high content of hydroxyl groups on its surface, favored the improved performance of the modified membrane.

When the incorporated GO had sheets with lateral dimensions contained mainly in the medium-sized interval (GO2, 1200–2300 nm) and small-sized interval (GO3, 500–1200 nm), the desalination performance with respect to permeate flux and salt rejection was detrimentally affected. The presence of a high content of medium sheets in GO2 affected the hydrophilicity of the membrane surface, producing an unfavorable impact on water flux. In addition, the use of materials with small sheets such as GO3 does not favor desalination performance as long as the stacking of sheets is not controlled. 

This work demonstrates that the use of multidimensional GO sheets promotes a synergist effect through the presence of large- and small-size GO sheets, in addition to the influence of their specific oxygenated surface group, balancing the membrane performance on both anti-biofouling and desalination properties modified during the IP process.

## Figures and Tables

**Table 1:** Percentages of population size distribution for used GOs.

Group	Classification	Size Range (nm)	Lateral Dimension Width			Lateral Dimension Length		
			GO1 (%)	GO2 (%)	GO3 (%)	GO1 (%)	GO2 (%)	GO3 (%)
1	Small	500–1200	74	88	100	48	12	100
2	Medium	1200–2300	18	12	-	28	88	-
3	Large	2300–3600	8	-	-	24	-	-

**Table 2:** X-ray Spectroscopy (XPS) analysis of the C1s and O1s signals of GO1, GO2, and GO3.

Sample	C1s			O1s			Atomic Ratio C/O
	Binding Energy (eV)	Groups	Area (%)	Binding Energy (eV)	Groups	Area (%)	
**GO1**	284.5	C–C/C=C	3.2	531.3	C–OH	23.3	2.09
	285.4	C–O	45.7	532.7	C=O	76.7	
	287.2	C=O	38.6				
	288.7	O-C=O	12.5				
**GO2**	284.5	C–C/C=C	13.2	531.6	C–OH	38.6	1.96
	285.4	C-O	26.1	532.7	C=O	34	
	286.8	C–O-C	45.9	533.1	COOH	27.4	
	288.7	O–C=O	14.8				
**GO3**	284.5	C–C/C=C	14.87	531.6	C–OH	80	1.77
	285.6	C–O	22.95	532.9	C=O	20	
	286.7	C–O–C	50.55				
	289	O–C=O	11.63				

**Table 3:** Surface properties of the membranes.

Membrane	Roughness (nm)	Contact Angle (°)
PA-PS	26.1	68 ± 2
PA+GO1-PS	77.9	56.7 ± 0.5
PA+GO2-PS	39.7	67 ± 10
PA+GO3-PS	87.1	23.7 ± 0.1

**Table 4:** Relative atomic concentrations and crosslinking degree of aromatic polyamide thin-film composite (TFC) membranes.

Membrane	Carbon (atom %)	Nitrogen (atom %)	Oxygen (atom %)	Ratio O/N	Ratio N/O	* CL (%)
PA-PS	73.8	11.7	13.5	1.1	0.86	78.5
PA+GO1-PS	70	14.1	15.9	1.1	0.89	82.5
PA+GO2-PS	70.2	13.4	16.4	1.2	0.82	70.3
PA+GO3-PS	71.8	13.4	14.8	1.1	0.91	85.7

* CL (%), Crosslinking degree calculated using the Equations (2) and (3).

**Table 5:** Anti-biofouling effect of membranes.

Membrane	Bactericidal Effect (%)	Anti-Adhesion Effect (%)
PA-PS	15	-
PA+GO1-PS	69	49
PA+GO2-PS	31	77
PA+GO3-PS	55	58

**Table 6:** Zeta potential of GOs used to modify membranes.

Material	Z Potential Range (mv) *
GO1	(−1)–(0)
GO2	(−45)–(−40)
GO3	(−50)–(−45)

* pH interval = (7–9).

**Table 7:** Desalination performance of membranes.

Membrane	Flux (L m^−2^ h^−1^)	Reject (%)
PA-PS	11 ± 1	93 ± 1
PA+GO1-PS	26 ± 3	90 ± 6
PA+GO2-PS	2 ± 1	91.5 ± 0.2
PA+GO3-PS	5 ± 1	83 ± 3

## Supplementary Materials

The following are available online at https://www.mdpi.com/2073-4360/12/12/2860/s1, Figure S1: CFU images for PA-PS membrane, Figure S2: CFU images for PA+GO1-PS membrane, Figure S3: CFU images for PA+GO2-PS membrane, Figure S4: CFU images for PA+GO3-PS membrane, Figure S5: XRD spectra of membrane synthesized, Figure S: Cross-section SEM of the membranes. (A,B) PA-PS; (C,D) PA+GO1-PS; (E,F) PA+GO2-PS; (G,H) PA+GO3-PS, Table S1: Number of counted colonies and CFU calculated, Table S2: Number of attached cells in the membrane surface obtained by fluorescence microscopy, Table S3: Polyamide thickness of the synthesized membranes.
