# Supplementary material for: Influence of Multidimensional Graphene Oxide (GO) Sheets on Anti-Biofouling and Desalination Performance of Thin-Film Composite Membranes: Effects of GO Lateral Sizes and Oxidation Degree
Source: Polymers (Basel). 2020 Nov 30;12(12):2860. doi: 10.3390/polym12122860 (PMC7760862; doi:10.3390/polym12122860)
Supplement: Supplementary file 1 [file polymers-12-02860-s001.pdf]

Supporting Information

Influence of Multidimensional Graphene Oxide (GO) Sheets on Anti-Biofouling and Desalination Performance of Thin-Film Composite Membranes: Effects of GO Lateral Sizes and Oxidation Degree

Bárbara E. Rodríguez [1], María Magdalena Armendariz-Ontiveros [2], Rodrigo Quezada [1], Esther A. Huitrón-Segovia [3], Humberto Estay [1], Alejandra García García [3,*] and Andreina García [1,*]

[1] Advanced Mining Technology Center (AMTC), Universidad de Chile, Av. Tupper 2007, 8370451 Santiago, Chile
[2] Departamento de Ciencias del Agua y Medio Ambiente, Instituto Tecnológico de Sonora, 5 de Febrero 818 Sur, Cd. Obregón, 85000 Sonora, CP, Mexico
[3] Group of Synthesis and Modification of nanostructures and Bidimensional Materials, Centro de Investigación en Materiales Avanzados S.C. Parque PIIT, 66628 Apodaca, NL, Mexico
* Correspondence: alejandra.garcia@cimav.edu.mx (A.G.G.); andreina.garcia@amtc.cl (A.G.); Tel.: +52-811156-0835 (A.G.G.); +56-22971015 (A.G.)

S1.- Bactericidal test

S1.1.- Bactericidal Effect

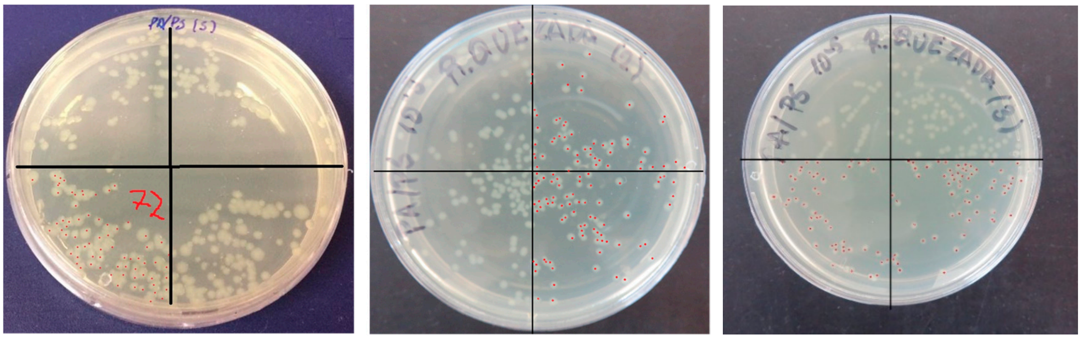

Figure S1. CFU images for PA-PS membrane.

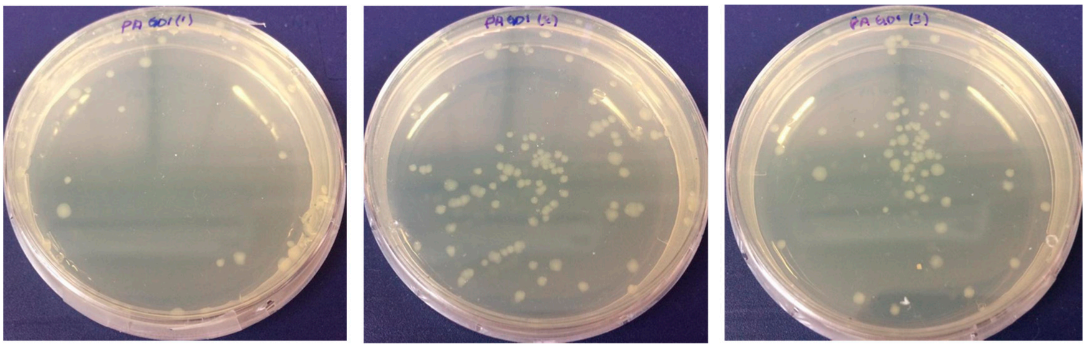

Figure S2. CFU images for PA+GO1-PS membrane.

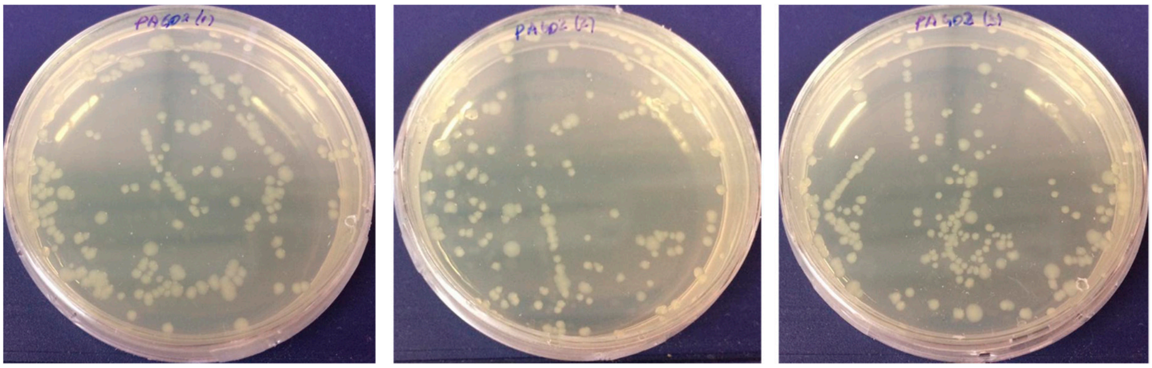

Figure S3. CFU images for PA+GO2-PS membrane.

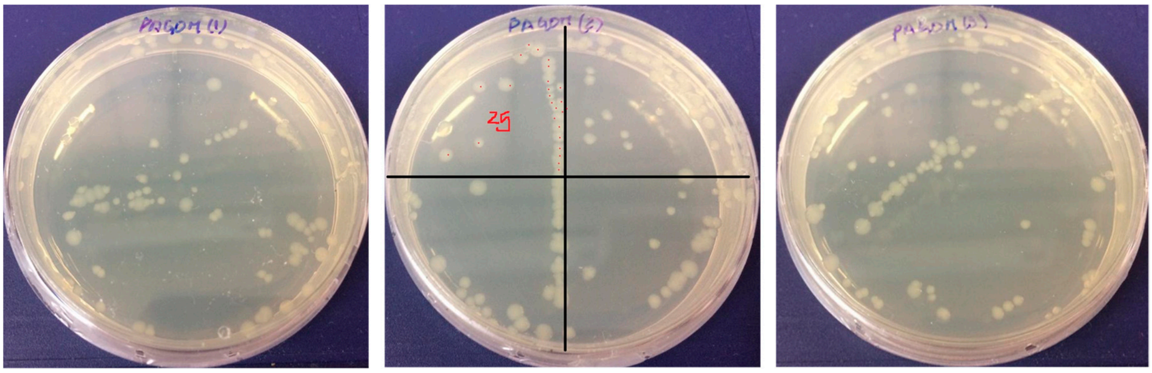

Figure S4. CFU images for PA+GO3-PS membrane.

The number of counted colonies on each plate for each experiment is shown in Table S1.

Table S1. Number of counted colonies and CFU calculated.

	Number of counted colonies			CFU average (cel/mL)× 10^{11}
**Experiment**	#1	#2	#3	
**PA/PS**	248	230	288	5.36
**GO1**	29	89	67	1.23
**GO2**	162	112	212	2.74
**GO3**	75	100	96	1.87

The eq. 1 was used to determine the amount of bacteria.

$$\#Bacteria = \#Number\ of\ colonies \times (dilution)^{-1} \times (inocolum)^{-1}(L) \quad (1)$$

In these experiments, the dilution used corresponds to 1×10^{-5} and the volume inoculated was equal to 50μL. A model calculation to the bacteria number for the GO3 experiment is shown as follows:

$$\#Bacteria(GO3-1) = 75 \times (10^{-5})^{-1} \times (50 \cdot 10^{-6})^{-1} = 1.50 \cdot 10^{11} \left(\frac{cel}{mL}\right) \quad (2)$$

$$\#Bacteria\ (GO3-2) = 100 \times (10^{-5})^{-1} \times (50 \cdot 10^{-6})^{-1} = 2.00 \cdot 10^{11} \left(\frac{cel}{mL}\right) \quad (3)$$

$$\#Bacteria\ (GO3-3) = 96 \times (10^{-5})^{-1} \times (50 \cdot 10^{-6})^{-1} = 1.92 \cdot 10^{11} \left(\frac{cel}{mL}\right) \quad (4)$$

Finally, the average between the values in eq. 2-4 is obtained:

$$\#Bacteria\ (GO3) = 1.87 \cdot 10^{11} \left(\frac{cel}{mL}\right) \quad (5)$$

S1.2.- Antiadhesion Effect

In the table S2 is shown the total number of attached bacteria determined with epifluorescence microscopy using the LIVE/DEAD viability kit. These data were used for making the calculation of anti-adhesion effect. The anti-adhesion effect was estimated respect to unmodified membrane according to the equation 6.

$$Antiadhesion\ effect\ (\%) = \left(\frac{(\textbf{\#Attached cells/mm2 in PA} - \textbf{PS}) - (\textbf{\#Attached cells/mm2 in modified membrane})}{(\textbf{\#Attached cells/mm2 in PA} - \textbf{PS})}\right) * 100\% \quad (6)$$

Table S2. Number of attached cells in the membrane surface obtained by fluorescence microscopy.

Experiment	#Attached cells /mm^2
**PA-PS**	426.92
**PA+GO1-PS**	216.67
**PA+GO2-PS**	97.59
**PA+GO3-PS**	181.00

S2.- DRX of the membranes

X-ray diffraction (XRD) of the membranes were obtained using Cu-Kα1 radiation of 1.54059 Å on a PANalytical Empyrean diffractometer (PANalytical Inc., Massachusetts, United States).

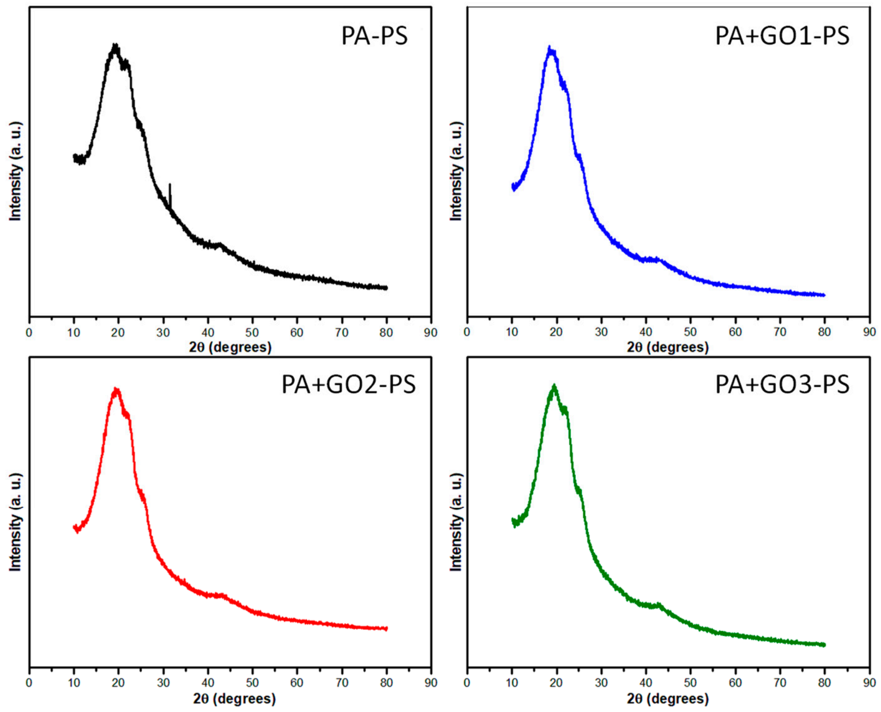

Figure S5. XRD spectra of membrane synthesized.

S3.- Cross-section SEM of the membranes

In order to estimate the polyamide layer thickness, the cross-sectional of the membranes was studied using scanning electron microscopy FE-SEM (Inspect F50, FEI) High Resolution Scanning Electron Microscope, Model INSPECT-F50, Thermo Fisher Scientific (FEI, Holanda). The membranes were then sputter coated with a thin film of gold (10 nm) to make them conductive. The coating was made using Sputter Coater Cressington TEDPELLA, model 108 with thickness controller MTM 20 Cressington. The membranes were snapped under liquid nitrogen to give a generally consistent and clean cut.

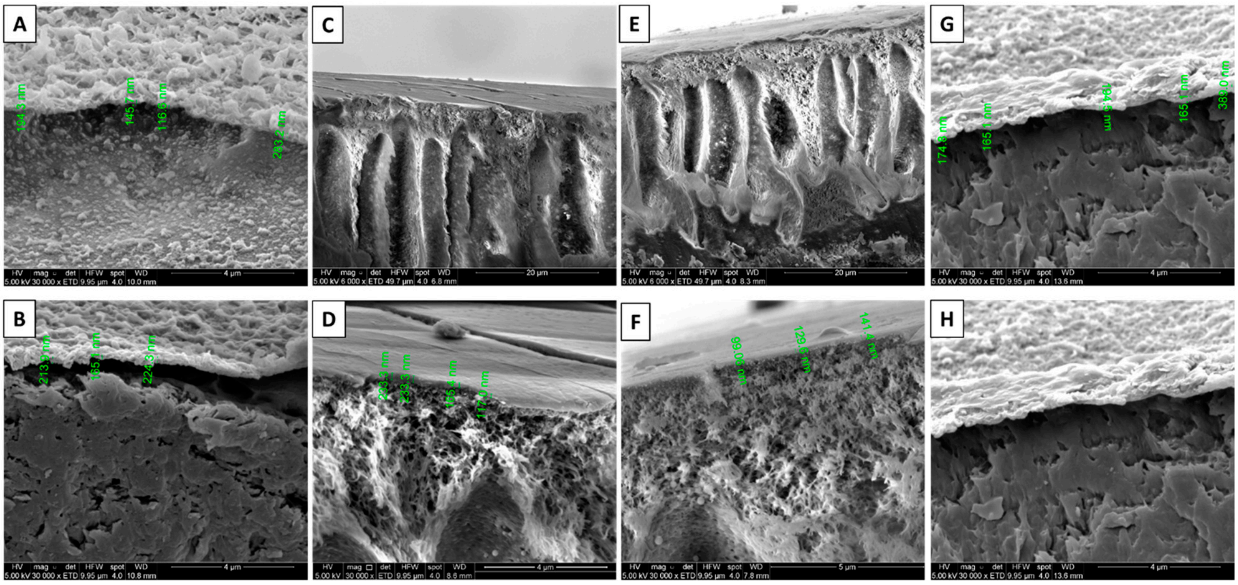

Figure S7. Cross-section SEM of the membranes. A-B) PA-PS; C-D) PA+GO1-PS; E-F) PA+GO2-PS; G-H) PA+GO3-PS.

Table S3. Polyamide thickness of the synthesized membranes.

Membrane	Thickness (nm)
**PA-PS**	192 ± 55
**PA+GO1-PS**	187 ± 57
**PA+GO2-PS**	123 ± 22
**PA+GO3-PS**	218 ± 97
